# Primary pulmonary mucosa-associated lymphoid tissue lymphoma computed tomography findings: a case report

**DOI:** 10.4076/1757-1626-2-6329

**Published:** 2009-07-06

**Authors:** Isabella Guedes Santos, Edson Marchiori, Gláucia Zanetti, Claudia Mauro Mano, Branca Sarcinelli-Luz, Flávia Gavinho Vianna, Juliana França Carvalho, Carla Assed, Alair Augusto SMD Santos, Alberto Domingues Vianna

**Affiliations:** Department of Radiology, Faculty of Medicine, Fluminense Federal UniversityRio de JaneiroBrazil

## Abstract

Primary pulmonary non-Hodgkin's lymphoma is a very rare neoplasm. It is most frequently represented by the mucosa-associated lymphoid tissue type. We describe a case of a 67-year-old woman who presented with fatigue, mild dyspnea, and consolidation on chest radiograph. The diagnosis of non-Hodgkin's lymphoma was established by transbronchial biopsy, and chemotherapy was then started. The patient was treated with 6 cycles of chemotherapy. She had good response to therapy and no progression of the disease was observed during a 4-year follow-up period. The clinical, radiological and histopathological features are described. This entity has an indolent course, good response to therapy and favorable prognosis. However, despite being an incidental radiological finding in a third of cases, this disease remains highly underdiagnosed.

## Introduction

Lymphomas of the lung can be classified into four categories of disease: primary pulmonary involvement, recurrent or secondary lymphoma of the lung, lymphoma in patients with post-transplantation lymphoproliferative disorders, and AIDS-related lymphoma [1-4].

Primary pulmonary lymphoma (PPL) is a rare entity, accounting for only 0, 5-1% of all pulmonary malignancies and less than 1% of all lymphomas. It is defined as a clonal lymphoid proliferation affecting one or both lungs (parenchyma and/or bronchi) in a patient with no evidence of mediastinal adenopathy, no detectable extrapulmonary involvement by clinical staging work-up, no past history of lymphoma, and no evidence of extrathoracic disease up to 3 months [1,5,6]. PPL is usually a non-Hodgkin lymphoma (NHL), and according to the World Health Organization's classification system, it can be divided into two types: mucosa-associated lymphoid tissue (MALT) and non-MALT. The former is represented by the low-grade extranodal marginal zone B-cell lymphoma (58%-87%), while the latter consists of diffuse large B-cell or high-grade lymphoma (11%-19%) and anaplastic large cell lymphoma [6-8]. MALT is a term that describes the specialized lymphoid tissue involved in mucosal defense, which is located beneath the epithelium [1,9].

When located in the lung, this lymphoma appears to arise from preexistent resident bronchus-associated lymphoid tissue (BALT) and is thought to be acquired as a result of chronic antigenic stimulation such as smoking, autoimmune diseases, or infections [1,6,7,9-11]. In fact, before the development of more accurate immunohistochemical techniques and molecular biology-based method, most cases were classified as pseudo-lymphomas, a benign inflammatory lymphocytic infiltrate [1,7].

Primary pulmonary MALT lymphoma often occurs in the fifth to seventh decade of life; subjects under the age of 30 years are rarely affected. The majority of studies reports that the two genders are equally affected [1,7], although a female predominance has been suggested [1]. They infiltrate the pulmonary interstitium, often massively, and may form nodular aggregates and extend into the air spaces [10]. Most of the patients (50%) are asymptomatic and the lesion is discovered incidentally on chest radiograph, while 46% have respiratory symptoms and nonspecific pulmonary complaints. In a minority of patients, constitutional features like weight loss, fever or malaise have been reported at presentation [5,6,10].

Pulmonary lymphoma can present with different radiological manifestations, including single or multiple nodules, masses, infiltrates and consolidations. This suggests that any radiological abnormality of the lung parenchyma may be a lymphoma [6]. Several treatment options are available, including tumor resection, radiotherapy, surgery with adjuvant chemotherapy or chemotherapy alone. The treatment remains controversial but the prognosis is excellent [5-7,9].

## Case presentation

A 67-year-old Afro-American Brazilian woman was admitted with a 4-month history of fatigue and progressive dyspnea on exertion. She was diagnosed with hepatitis C three months earlier, without treatment. She denied fever, weight loss, smoking and drug or alcohol abuse. Physical examination revealed an ill-looking, emaciated woman, with a blood pressure of 120/80 mmHg, a heart rate of 100 bpm, and a respiratory rate of 27 breaths/min. In the assessment of the respiratory system, there were signs of airspace consolidation in the upper third of the left hemithorax. Inspiratory crackles were heard in the lower third of the hemithorax. The remainder of the examination was normal.

Laboratory evaluation revealed a red blood cell (RBC) count of 5, 13 million/mm³, hemoglobin level of 11.8 g/dl, hematocrit of 35.9%, and platelet count of 117,000/mm³. The WBC count was 3,600/mm³ (basophils 1%; eosinophils 3%; band neutrophils 0%; segmented neutrophils 56%; lymphocytes 34%; monocytes 6%); MCV 73 µm³; MCHC 32.7 g/dl; ESR 53 mm/h. Absolute and relative reticulocyte count were 320,450/mm³ and 6.5%, respectively. Hematoscopy showed anisocytosis, microcytosis and hypochromia. Liver function tests were normal, and serology for HIV was negative.

**Figure 1.**
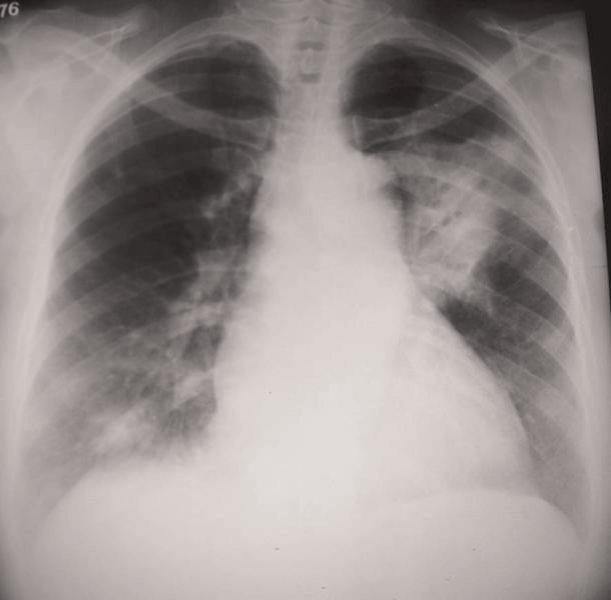
Chest X-ray shows a large consolidation with air bronchograms in the left lung, and a small area of consolidation in the lower region of the right lung.

**Figure 2.**
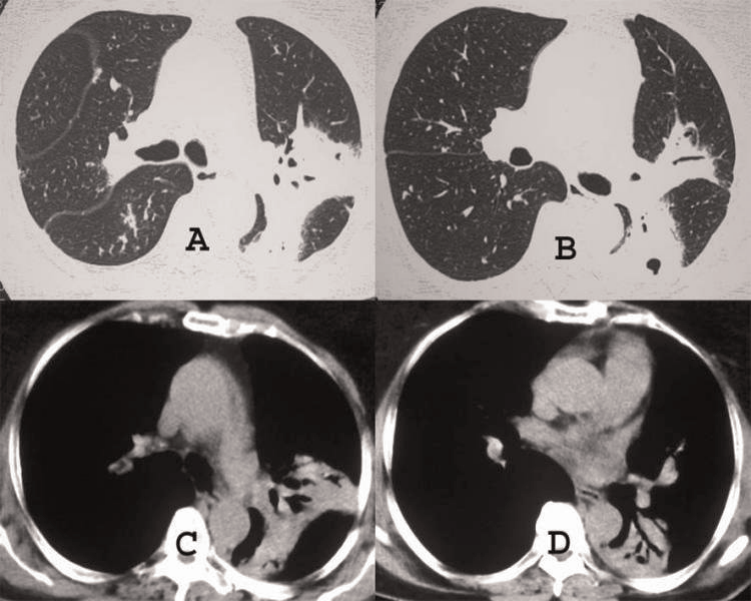
High-resolution CT scans with parenchymal (**A** and **B**) and mediastinal (**C** and **D**) window settings demonstrate a consolidation with air bronchogram in the apicoposterior segment of the left upper lobe, and in the superior segment of the left lower lobe.

Chest radiograph revealed consolidations in the upper lobe of the left lung and in the lower lobe of the right lung (Figure 1). High-resolution computed tomography (CT) showed a consolidation with air bronchograms in the apicoposterior segment of the left upper lobe and small areas of consolidation in the right lower lobe. No lymphadenopathy was detected (Figure 2).

She had a negative tuberculin skin test reaction, and six sputum samples were negative for acid-fast bacilli. Cultures for mycobacteria and fungi were also negative. Bronchoscopy showed no abnormalities, and the cultures of the bronchoalveolar lavage fluid were negative for tuberculosis, fungi and other microorganisms. Transbronchial biopsy revealed infiltration of atypical lymphoid B cells. Immunohistochemically, the lung tumor cells expressed immunoreactivity for anti-CD20, oncoprotein, Bcl-2, kappa light chain of immunoglobulin and cell proliferation antigen Ki-67 (<10%). Anti-cytokeratin pan antibody revealed areas of epithelial injury. Based on these findings, the diagnosis of low-grade B-cell non-Hodgkin's lymphoma (MALT lymphoma) was made.

Additionally, a bone marrow biopsy was performed and showed no neoplastic infiltration. Upper gastrointestinal endoscopy did not find areas of metaplasia, and abdominal CT was normal. The absence of lymphadenopathy or other signs of extrathoracic disease defined the lymphoma as primary and with exclusive pulmonary involvement. She received six cycles of chemotherapy with cyclophosphamide, vincristine and prednisone (Oncovir) over a three-month period, with complete remission of the symptoms. The patient underwent biannual clinical and radiological follow-up, and during the next four years, no changes were detected. There was no additional treatment performed, and the disease was considered in remission.

## Discussion

Due to its rarity, relatively little is known about the natural history of BALT lymphomas in terms of local infiltration and dissemination. However, it is characteristic that the lesions remain restricted to the lungs for long periods of time before dissemination. The interval between the first clinical or radiological manifestation and diagnosis ranges from 5 months to 8 years, which reflects the indolent course of this disease [1,7].

When present, symptoms such as cough, mild dyspnea, chest pain and occasionally hemoptysis, are nonspecific. Pulmonary auscultation reveals crackles in 20% of cases [1]. Our patient had fatigue and progressive dyspnea on exertion as unique symptoms, associated with nonspecific laboratory abnormalities and signs of lung consolidation on physical examination and in chest radiograph.

The most common radiological findings of PPL are uni- or bilateral pulmonary infiltrates or nodules, in a peribronchovascular distribution [2-4,6]. Usually the appearance is an area of localized alveolar opacity with poorly defined margins, which is associated in nearly 50% of cases with air bronchograms [1,10]. CT, which is more sensitive, usually demonstrates consolidations, areas of ground-glass opacities and peribronchovascular thickening. These lesions are usually bilateral (60-70%) and multiple (70-77%) [3,10,11].

The criteria for diagnosis of primary pulmonary lymphoma are not clearly established and include histology, immunohistochemistry and molecular biology findings, and the natural history of various specific types of lymphomas. An extensive staging workup, including upper and lower gastrointestinal endoscopies and bone marrow biopsies is also recommended [5].

Bronchial endoscopy usually shows a normal macroscopic aspect, although abnormalities ranging from mucosal inflammation to bronchial stenosis can be observed [1]. BAL fluid may demonstrate an increased cellularity with a predominant lymphocytosis, and flow cytometry analysis can aid in establishing the diagnosis. A histological diagnosis can be obtained by bronchial or transbronchial biopsy or by transthoracic fine-needle aspiration [8].

This diagnostic yield is higher when it targets visible endobronchial lesions or radiographic abnormalities; when it does not, a surgical biopsy is essential for establishing the diagnosis in some cases [5].

Treatment provides extremely low mortality rates, reaching a 5-year survival rate of 100% [9]. Although therapeutic consensus has not been clearly established, in asymptomatic cases with indolent clinical course, initial observation seems to be a reasonable option for some studies. However, other studies show that BALT lymphomas can occasionally progress more rapidly to massive lung infiltration, indicating that diagnostic efforts and treatment should not be delayed [6,7]. In most studies, surgical resection, either alone or in combination with chemotherapy, has been the mainstay of therapy. Despite their indolent behavior and excellent prognosis, the prognostic factors have not been well defined [5] and the disease tends to relapse frequently. Its progression has been associated with large-cell transformation of MALT lymphomas [7].

## Abbreviations

AIDS: Acquired immunodeficiency syndrome
BAL: Bronchoalveolar lavage
BALT: Bronchus-associated lymphoid tissue
CT: Computed tomography
CT: Computer tomography
ESR: Erythrocyte sedimentation rate
HIV: Human immunodeficiency virus
MALT: Mucosa-associated lymphoid tissue
MCHC: Mean corpuscular hemoglobin concentration
MSV: Mean corpuscular volume
NHL: Non-Hodgkin lymphoma
PPL: Primary pulmonary lymphoma
RBC: Red blood cell
WBC: White blood cells
